# Indications, Techniques and Future Perspectives of Walled-off Necrosis Management

**DOI:** 10.3390/diagnostics14040381

**Published:** 2024-02-09

**Authors:** Edoardo Troncone, Rosa Amendola, Fabio Gadaleta, Elena De Cristofaro, Benedetto Neri, Pasquale De Vico, Omero Alessandro Paoluzi, Giovanni Monteleone, Andrea Anderloni, Giovanna Del Vecchio Blanco

**Affiliations:** 1 Department of Systems Medicine, University of Rome “Tor Vergata”, 00133 Rome, Italy; edoardo.troncone@ptvonline.it (E.T.);; 2 Department of Anaesthesia, University of Rome “Tor Vergata”, 00133 Rome, Italy; 3 Endoscopy Unit, First Department of Internal Medicine, Fondazione IRCCS Policlinico San Matteo, University of Pavia, 27029 Pavia, Italy

**Keywords:** necrotizing pancreatitis, walled-off necrosis, EUS drainage

## Abstract

Necrotizing pancreatitis is a complex clinical condition burdened with significant morbidity and mortality. In recent years, the huge progress of interventional endoscopic ultrasound (EUS) has allowed a shift in the management of pancreatic necrotic collections from surgical/percutaneous approaches to mini-invasive endoscopic internal drainage and debridement procedures. The development of lumen-apposing metal stents (LAMSs), devices specifically dedicated to transmural EUS interventions, further prompted the diffusion of such techniques. Several studies have reported excellent outcomes of endoscopic interventions, in terms of technical success, clinical efficacy and safety compared to surgical interventions, and thus endoscopic drainage of walled-off necrosis (WON) has become a fundamental tool for the management of such conditions. Despite these advancements, some critical unresolved issues remain. Endoscopic therapeutic approaches to WON are still heterogeneous among different centers and experts. A standardized protocol on indication, timing and technique of endoscopic necrosectomy is still lacking, and experts often adopt a strategy based on personal experience more than robust data from well-conducted studies. In this review, we will summarize the available evidence on endoscopic management of WON and will discuss some unanswered questions in this rapidly evolving field.

## 1. Introduction

**Figure 1:**
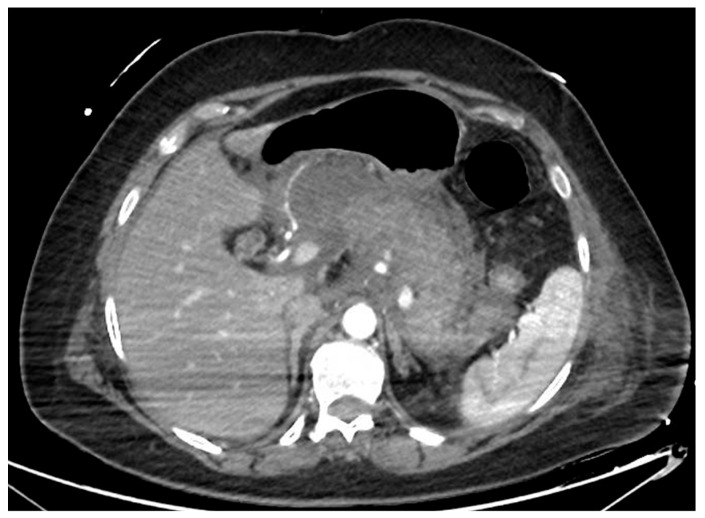
CT scan image of a necrotizing pancreatitis, with hypoenhancing areas of pancreatic parenchyma. CT, computed tomography.

Acute pancreatitis is one of the most common causes of hospitalization among gastroenterological diseases and accounts for relevant expenses for the healthcare system [1,2]. In 5–10% of cases, the acute inflammatory process leads to necrosis of the pancreatic parenchyma and/or peripancreatic tissue (Figure 1). Necrotizing pancreatitis has usually a more severe clinical course, more frequent local and systemic complications, organ failure, need for interventions and higher mortality rates [3]. According to the Atlanta classification, peripancreatic collections are classified depending on the timing from the acute onset of pancreatitis (Table 1). After 4 weeks, collections usually have definite limits and mature walls, and are called pseudocysts or walled-off necrosis (WON), depending on the absence or presence of solid necrotic material. Endoscopic drainage and debridement (i.e., endoscopic necrosectomy, EN) have gained great popularity for the management of WON, in light of its less invasive nature compared to percutaneous or surgical interventions, together with a high clinical efficacy. In this review, we will summarize the evidence available regarding the indication, timing and technique of endoscopic management of WON, including indication and technique of EN, and we will discuss some unresolved issues and future perspectives in this field.

## 2. Pancreatic Walled-off Necrosis: Indications for Drainage

**Figure 2:**
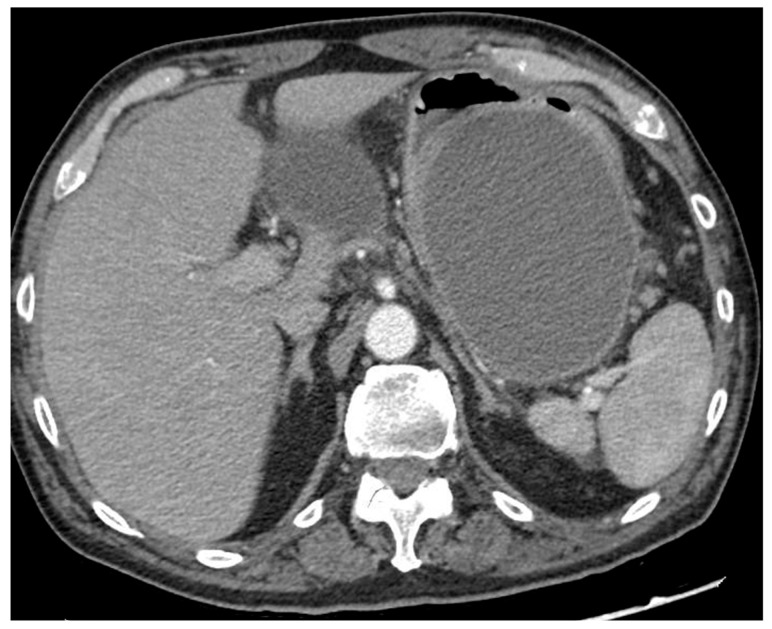
CT scan image of a large collection determining symptomatic gastric compression. CT, computed tomography.

Not all necrotizing pancreatitis need invasive treatment. The need for invasive approaches is limited to specific conditions and is mainly dictated by the clinical presentation. The main indication for invasive treatment is the infection of the necrosis, which can develop in about one-third of patients with necrotizing pancreatitis, with a mortality rate of up to 19% [3]. Infected pancreatic necrosis (IPN) can be suspected in the presence of radiological signs of infection (i.e., gas within the collection at cross-sectional images), or in case of persistent organ failure and/or lack of clinical improvement (i.e., fever, leukocytosis or persistent increase of inflammatory markers), despite optimal medical therapy. IPN can be proved by EUS-guided fine-needle aspiration (FNA) of the necrotic material and microbiological culture, but this step is not mandatory if the clinical suspect is high [4]. Indeed, data from a post hoc analysis in a Dutch prospective database of 639 patients with necrotizing pancreatitis showed that positive microbiological culture from FNA necrotic material was confirmed in 86% of cases in subsequent cultures from necrotic material collected during drainage, while the infection was confirmed in 80% of cases in which the diagnosis of IPN had been made clinically [5]. The most accurate sign of infection remained the presence of gas within the collection seen in cross-sectional images, which was confirmed at subsequent cultures in 96% of cases [5]. Therefore, FNA to prove IPN is currently limited to unclear and selected cases and should not be performed routinely, due to the high risk of false-positive and false-negative results [4]. Other indications for drainage are pain and organ compression by the collection, including gastric outlet obstruction and intestinal and biliary compression (Figure 2).

## 3. Which Is the Best Timing for Invasive Interventions?

Surgical experience showed that a too-early intervention on pancreatic necrosis often resulted in the worst outcome and increased risk of bleeding and other complications, due to the insufficient demarcation between necrotic and viable tissue, and therefore intervention should be delayed, when possible [6,7]. This concept has been confirmed also for minimally invasive drainage approaches. Indeed, a recent RCT from the Dutch Pancreatitis Study Group reported that immediate drainage was not superior compared to postponed drainage concerning the complication rate in patients with infected WON, and the delayed drainage strategy was overall associated with fewer interventions, including necrosectomy [8]. Strikingly, 35% of patients in the delayed-drainage group were eventually treated with antibiotics only, without the need for any invasive procedure, thus demonstrating that antibiotic efficacy was superior to what had been reported previously [9]. During the early phase of necrotizing pancreatitis, it is of paramount importance to optimize the conservative management, which include enteral feeding in patients who do not tolerate early oral feeding, and antibiotic therapy with antibiotics targeting gut-derived bacteria (e.g., carbapenems, quinolone, metronidazole or high-dose cephalosporins) if IPN is suspected [4]. These measures are aimed at reducing the risk of infection in patients with sterile necrosis and improving general conditions and nutritional status in patients who will need invasive interventions. From the endoscopist’s point of view, a necrotic collection should have mature inflammatory walls to be drained internally with an endoscopic transmural intervention, due to the risk of material leakage and/or perforation when treating an immature collection. Therefore, it is generally accepted to delay the endoscopic intervention for 4 weeks from the onset of pancreatitis, which is the time that it usually takes the acute pancreatic collection to mature in pseudocyst or WON, while in the case of urgent indications during the first 4 weeks, it is recommended to perform a percutaneous drainage [4]. However, the paradigm of the “4 weeks” for endoscopic drainage has been recently challenged by studies that reported good technical and clinical outcomes after “early” drainage of pancreatic collections, even though higher mortality rates and longer hospitalization have been also described [10,11,12]. In 2023, a meta-analysis performed by Ramai and colleagues aimed to evaluate specifically the clinical efficacy and safety of EUS-guided drainage of pancreatic fluid collections <4 weeks or ≥4 weeks after the acute event [13]. The authors included six studies with a total of 630 patients, in which 182 patients (28.9%) were enrolled in the early-drainage cohort and 448 patients (71.1%) in the standard-drainage cohort. No statistically significant differences were found in overall technical success, clinical success and adverse events. However, the early group showed a significantly longer hospital stay (23.7 vs. 16.0 days) [13]. These data indicate that endoscopic drainage of necrotic collection before 4 weeks is overall feasible, effective and safe. However, it should be noted that these data come from retrospective studies, and thus the quality is limited. Moreover, the “early” necrotic collections were drained in some cases after a mean time of about 3 weeks after acute pancreatitis, and such collections already showed a partially mature wall. Therefore, it is not possible to generalize these observations to very early or immature necrotic collections. Waiting for more solid data, the decision to endoscopically drain a necrotic collection before 4 weeks should be individualized.

## 4. Therapeutic Approaches to Walled-off Necrosis: From Open Surgery to Endoscopic Drainage

Once the need for drainage has been defined, the approach to be chosen depends on several factors. Timing from the acute onset of pancreatitis, as discussed above, is an important one. The general condition of the patient, characteristics of the collection and the expertise available at the center involved also have to be considered. Solid evidence has shown that a minimally invasive approach should be preferred over open surgical intervention, as it provides better outcomes with fewer adverse events (AEs). Indeed, open surgery to perform drainage and debridement of necrosis, in very sick patients, is burdened by a very high morbidity and mortality, while percutaneous and/or mini-invasive surgical approaches are very effective [3,14,15,16,17,18,19,20]. In 2010, a randomized controlled trial (RCT) from van Santvoort and colleagues first reported the superiority in terms of major complications of a step-up approach from percutaneous drainage to retroperitoneal surgical debridement compared to upfront surgery in IPN [18]. This study strongly supported the concept of a minimally invasive approach and suggested that more invasive interventions should be limited to patients who do not respond properly to initial management. Indeed, patients seem to benefit more from the control of the source of infection, which is provided by the drainage, rather than the complete (and early) removal of the infected necrotic tissue, which was the aim of the open necrosectomy. This concept was further confirmed in a large cohort study [19]. Later on, endoscopic drainage techniques challenged the percutaneous approach as a minimal invasive intervention. The RCT from van Brunschot et al. investigated whether an endoscopic step-up approach was effective in treating infected WON compared to the “standard” surgical step-up approach [20]. The study included 98 patients, randomized to endoscopic (*n* = 51) or surgical (*n* = 47) step-up intervention. The endoscopic step-up protocol consisted of endoscopic ultrasound (EUS)-guided transmural drainage, followed by direct endoscopic necrosectomy in case of unsatisfactory clinical response. The primary endpoint was again a composite of major complications or death during 6-month follow-up. The primary endpoint occurred in 22 of 51 patients (43%) in the endoscopy group and in 21 of 47 patients (45%) in the surgery group (risk ratio [RR] 0.97, 95% CI 0.62–1.51; *p* = 0.88). Similarly, mortality did not differ between groups (18% vs. 13%, R 1.38, 95% CI 0.53–3.59, *p* = 0.50). Notably, the endoscopic group showed a significantly lower rate of pancreatic fistula (5% vs. 32%), shorter hospitalization length and lower mean total costs per patient, compared to the surgical group. Therefore, the study did not demonstrate the superiority of the endoscopic approach but showed advantages in some secondary endpoints [20]. In contrast, the RCT from the US group of Bang et al. reached different conclusions [21]. In this study, patients were randomized using either a surgical mini-invasive step-up approach (i.e., laparoscopic cystogastrostomy or video-assisted retroperitoneal debridement (VARD), depending on the location of the collection; *n* = 32) or an endoscopic step-up approach (*n* = 34). The primary endpoint (a composite of major complications or death) was met in 11.8% of patients in the endoscopy group and in 40.6% of patients in the minimally invasive surgery group (RR 0.29; 95% confidence interval 0.11–0.80; *p* = 0.007). This difference was statistically significant and mainly related to the difference between groups in pancreatic fistulas (enteral or cutaneous) in the surgical group (0% vs. 28.1%, *p* = 0.001). Importantly, the mean number of major complications per patient was significantly higher for surgery compared with endoscopy, as well as the costs, and the quality-of-life scores were higher for endoscopy [21]. A meta-analysis including three RCTs on endoscopy vs. mini-invasive surgery for WON management (182 patients) confirmed these results, reporting that new-onset multiple-organ failure, enterocutaneous fistula/perforation and pancreatic fistula were significantly lower for endoscopic interventions compared to surgery, while the length of hospital stay was significantly shorter for endoscopy [22]. Overall, the mortality did not differ between groups (14.5% vs. 16.1%) [20,21,22,23]. These data boosted the use of endoscopy to manage pancreatic necrotic collection, and the endoscopic step-up approach should be adopted if the expertise is locally available [4,24,25,26,27].

## 5. EUS-Guided Drainage of Walled-off Necrosis: Technical Aspects and Clinical Outcome

**Figure 3:**
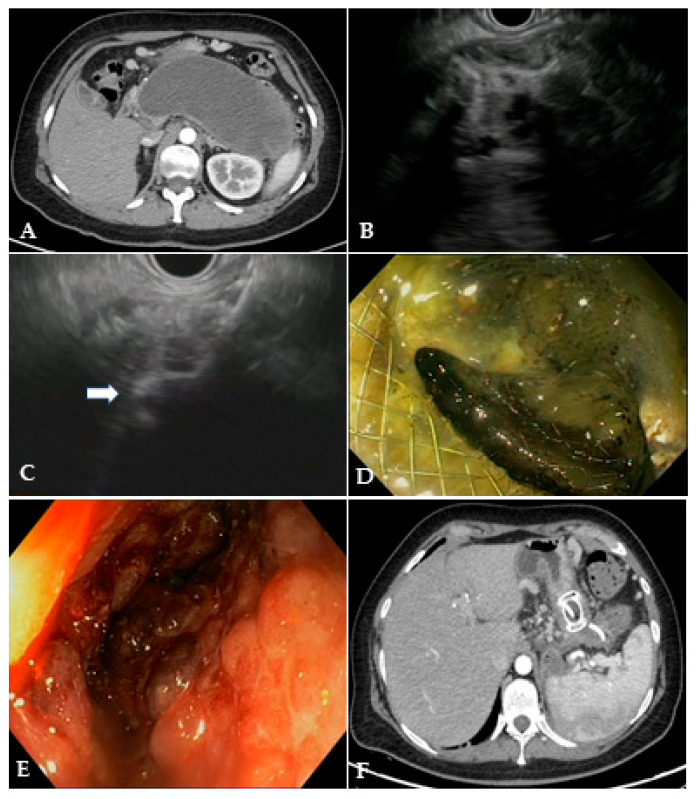
(**A**) CT scan and (**B**) EUS image of a large WON with large amount of solid necrotic material; (**C**) EUS image of the distal flange of a 20 mm LAMS (white arrow) deployed into the WON; (**D**) endoscopic image through the LAMS of the necrotic cavity completely filled with necrotic debris; (**E**) endoscopic image of the clean cavity after drainage and repeated necrosectomy sessions; (**F**) CT scan image of the collapsed cavity with indwelling LAMS and a 10 French/4 cm coaxial DPS. CT: computed tomography; EUS: endoscopic ultrasound; WON: walled-off necrosis; LAMS: lumen-apposing metal stent; DPS: double-pigtail stent.

**Figure 4:**
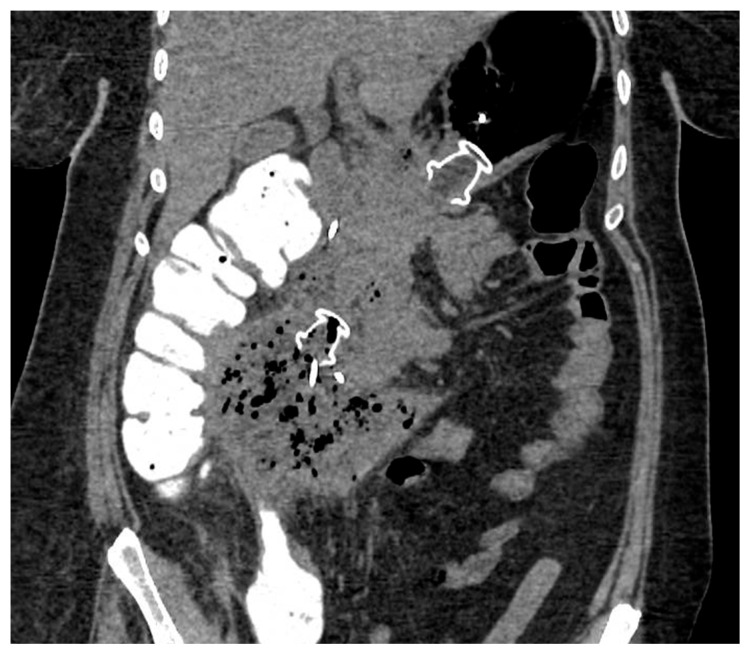
CT scan image of WON drained endoscopically with multiple transluminal gateway technique, with a transgastric 20 mm and a transduodenal 15 mm LAMS. CT: computed tomography; WON: walled-off necrosis; LAMS: lumen-apposing metal stent.

EUS-guided drainage is usually performed with curvilinear therapeutic EUS scopes, which are provided with a 3.7 mm working channel. The use of an EUS scope instead of a standard endoscope allows for precisely localizing the collection, especially when the collection does not produce a bulging within the gastric or duodenal lumen, as well as the relations with contiguous anatomical structures and the presence of intervening vessels. Therefore, EUS-guided drainage should be the preferred method of endoscopic drainage [4]. After locating the target collection, the optimal site of drainage is identified, which usually corresponds to the closest point to the gastrointestinal wall, whether it is in the stomach or duodenum. However, when choosing the site of EUS access, one should pay attention to the possible need for future transmural interventions, as too proximal or distal stenting in the gastric body could make subsequent endoscopic access to the collection more difficult. Traditionally, EUS-guided drainage was performed with a multi-step process with fluoroscopic assistance, which usually involved the EUS-guided access to the collection with a 19 Gauge FNA needle, the insertion of a 0.025- or 0.035-inch guidewire, dilation of the tract with a cystotome and balloon, and placing the stents for the cystogastrostomy or cystoduodenostomy. Initially, stents with luminal indication (i.e., biliary stents) were adapted to transmural drainage, such as plastic double-pigtail stents (DPSs) or self-expanding metal stents (SEMSs), biliary or esophageal. The advantages of using SEMSs compared to DPSs rely on the larger caliber, which theoretically allows better drainage of solid necrotic material and easier access to the collection through the stent, as well as on the expanding force and the covering of the meshes that seal the tract, minimizing the risk of leakage of material. Several studies have investigated the efficacy and safety of WON drainage with DPSs or SEMSs, reporting a high rate of clinical success [28,29,30]. However, SEMSs are particularly prone to migration due to non-dedicated tubular design, and they may have a higher risk of bleeding secondary to the traumatism of the long tubular ends of the stent. EUS-guided drainage has been revolutionized by the creation of dedicated stents, namely lumen-apposing metal stents (LAMSs). LAMSs are fully covered, “dumb-bell”-shaped braided nitinol metal stents, with wide anti-migratory flanges, that provide effective lumen-to-lumen apposition and large caliber (up to 20 mm) to drain solid necrosis. In addition to these features, the delivery system with an incorporated cautery tip (“hot” LAMSs), which allows direct access to the target in a “free-hand” fashion without a pre-inserted guidewire, has significantly simplified the drainage technique, helped avoid the drawbacks of the multi-steps procedure and further favored the diffusion of the EUS-guided technique. WON drainage with LAMSs showed an excellent technical and clinical success rate and has rapidly become the standard of care for EUS-guided WON drainage (Figure 3) [31,32,33]. Complex WON, mainly due to the size and/or extension of the collection, may need multiple transgastric or transduodenal access to be effectively drained (Figure 4). In 2011, Varadarajulu and colleagues described the multiple transluminal gateway technique (MTGT), reporting retrospectively the outcome of 12 patients with symptomatic WON treated with this technique, compared to 48 patients treated with conventional single-tract drainage [34]. In the MTGT group, one tract was used to flush normal saline solution via a nasocystic catheter, while multiple stents were placed in the other tracts to facilitate the drainage of necrotic material. After adjusting for the size of the WON and pancreatic stenting, the study showed that patients treated by MTGT had better clinical outcomes and less need for surgery compared with the conventional-drainage group [34]. Similarly, some collections may benefit from a combined endoscopic and percutaneous drainage, for example due to extension to abdominal sites far from the gastrointestinal tract and not amenable for endoscopic drainage, such as the pelvic paracolic gutters [4]. This “dual modality” drainage is clinically effective and reduces the length of hospitalization and endoscopic interventions [35]. The indication and timing of the MTGT approach are still unclear. The guidelines from the European Society of Gastrointestinal Endoscopy (ESGE) suggest additional tract drainage in case of very large WON (i.e., >12 cm), or insufficient response to the first drainage, thus a step-up approach [4]. However, single-step MTGT with LAMSs has been also reported for complex WON with good results, although in a small cohort of patients [36]. Currently, solid data about the selection of patients who may benefit from MTGT from the index drainage procedure (that is, from the first procedure) are lacking, and prospective studies are awaited.

## 6. Plastic Stents or Lumen-Apposing Metal Stents for EUS-Guided Drainage?

Clinical advantages of LAMSs over DPSs for WON drainage have been highlighted by several observational retrospective studies [32]. Furthermore, larger LAMS caliber seems to be associated to improved drainage. A retrospective study including 306 patients investigated the outcome of WON drainage using a 20 mm LAMS, in comparison with a 15 mm LAMS, and found that the former was associated with a lower number of necrosectomy sessions, even though the overall success rate was similar [37]. Nonetheless, when the superiority of such stents has been challenged in RCT, the results have become less obvious. The first RCT comparing DPSs and LAMSs in symptomatic WON was published by Bang and colleagues in 2019 and included 60 patients randomized to LAMS (*n* = 31) or DPS (*n* = 29) placement [38]. The primary outcome was the total number of procedures to achieve treatment success, and this did not differ between groups (median 2, range 2–7 in the LAMS group vs. 3, range 2–7, in the DPS group, *p* = 0.192). Moreover, no differences were found in treatment success, AEs, total number of procedures for clinical success, readmissions, length of hospital stay and overall treatment costs between cohorts. However, a significant stent-related adverse-events rate was observed after 3 weeks of the index intervention in the LAMS cohort, and this was mainly related to bleeding events (see below). This observation led to a change in the study protocol, and the LAMS was removed after 3 weeks if the WON had resolved. The only parameter that favored LAMSs over DPSs was the procedure time, which was significantly shorter in the LAMS groups (15 vs. 40 min, *p* < 0.001) [38]. After the publication of these results, an intense debate among interventional endoscopists started about the optimal timing of LAMS removal to avoid severe adverse events, and the strategy of LAMS removal after 4 weeks became widely accepted. In 2022, Karstensen and colleagues reported data from a single-center RCT comparing DPSs and LAMSs in large WON (>15 cm) [39]. This study focused on a sub-group of complex WON, using the largest LAMS available so far (i.e., 20 mm), and aimed to evaluate the number of necrosectomies needed to achieve clinical success. Forty-two patients were randomized (22 in the DPS group, 20 in the LAMS group), and no differences were found in technical and clinical success rate, as well as in the mean number of necrosectomies (2.2 for DPS, 3.2 for LAMS; *p* = 0.42). Again, LAMSs were not proven to be superior to DPSs in WON treatment. However, it should be noted that, according to the study protocol, the DPS groups underwent a weekly procedure of tract dilation, which is not a common strategy and may have improved the outcome in such a group [39]. Boxhoorn et al. performed a comparative non-randomized study using a prospective cohort of 53 patients who underwent LAMS placement for infected WON and compared this cohort with the DPS group from the TENSION trials [20,40]. The primary endpoint (the need for endoscopic necrosectomy) did not differ between groups (64%, *n* = 34 in the LAMS groups; 53%, *n* = 27 in the DPS group). Similarly, the secondary endpoints (mortality, major complications, hospital stay and healthcare costs) were not statistically different between the two groups. A recent meta-analysis of RCT including 206 patients further strengthened these findings and confirmed that LAMSs are not superior to DPSs for WON management regarding the main technical and clinical outcomes [41]. Data from these studies are summarized in Table 2. These data challenge the most diffused beliefs about LAMSs, as the large and fixed caliber of such stents seems not to increase clinical success, does not prevent occlusion and AEs, and does not reduce the need for endoscopic necrosectomies. So, is this the end of the story? Probably not. First, as stated above, the aggressive drainage protocol reported in the DPS group in the Karstensen study may not reflect the usual practice in these cases. Moreover, three patients experienced relevant adverse events in this group after dilation of the tract (two retroperitoneal perforation; one sepsis). Therefore, the safety of this approach is to be demonstrated, while the fixed diameter of the LAMSs avoids the need for repeated dilation, making the access to the cavity easier for direct necrosectomy. Second, the advantages of a short procedural time could be relevant in cases of sick patients with severe sepsis, thus favoring fast drainage with LAMSs in these cases. Costs also are a relevant aspect when treating these patients. Indeed, LAMSs have a higher cost compared to the cheaper plastic stents, and this could be interpreted as an argument in favor of DPSs, as the index procedure with LAMSs is more expensive. However, when comparing the overall costs of the treatment, comparative studies showed that there are not significant differences between LAMSs and DPSs, indicating a minor impact of the LAMS cost on the overall expenses of the management of WON [38,39,40]. Instead of defining a fixed protocol that fits every patient, it seems important to individualize the treatment (and the stent) to each clinical scenario. More robust data are awaited on this topic.

## 7. Direct Endoscopic Necrosectomy: When and How to Perform It

**Figure 5:**
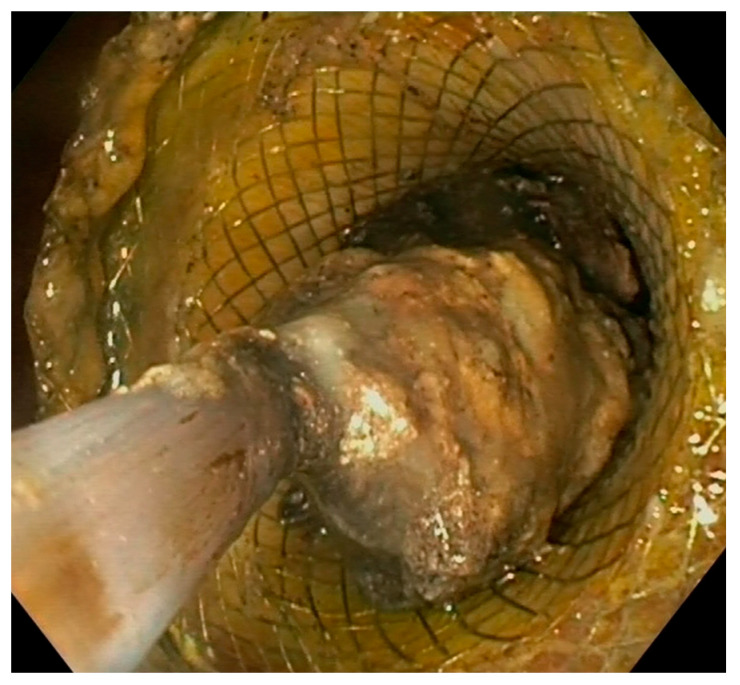
Endoscopic view of direct necrosectomy through a 20 mm LAMS with a polypectomy snare. LAMS: lumen-apposing metal stent.

**Figure 6:**
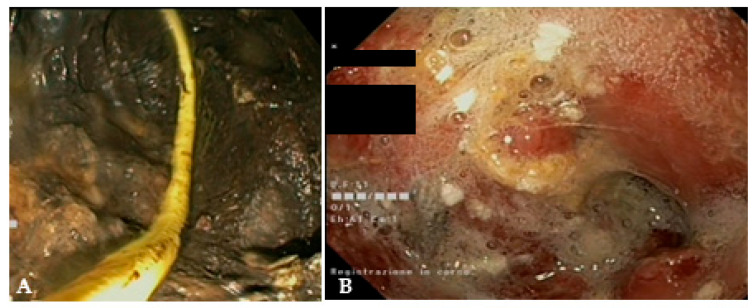
(**A**) Endoscopic view of a naso-cystic catheter within a necrotic collection; (**B**) endoscopic view of a retroperitoneal cavity after lavage with peroxide hydrogen.

Endoscopic necrosectomy is a complex, labor-intensive, time-consuming procedure and is a critical part of WON management. Despite the huge advancements achieved in endoscopic drainage techniques in recent years, necrosectomy still largely depends on non-dedicated devices and is performed with significant variability in technique and timing among centers [42]. Even experts are doubtful about the best way to perform it [43,44]. Direct endoscopic necrosectomy is performed after access within the cavity with a forward-viewing endoscope, usually after dilation of the mature fistula or through the LAMS, which can be also balloon dilated, if necessary. Some authors have suggested that removing the LAMS and performing direct necrosectomy through the naked fistula could improve scope maneuverability and extraction of larger pieces of necrosis [45]. After removal, the same LAMS can be safely repositioned at the end of the procedure, if drainage and additional interventions are still needed [45,46,47]. A therapeutic gastroscope allows better suction of liquid debris due to the larger working channel, and a distal attachment cap helps the mechanical removal of solid material. It is mandatory to use CO_2_ insufflation to reduce the risk of air embolism [4]. The first question is what is the optimal timing to perform endoscopic necrosectomy? A widespread strategy is to perform an endoscopic necrosectomy if initial drainage provides an insufficient clinical response. Surgical and endoscopic step-up studies demonstrated that minimally invasive interventions lead to clinical success in a relevant proportion of patients; thus, more invasive strategies are justified only in selected cases [18,20,21,48]. Indeed, about 30–50% of patients with WON undergoing endoscopic treatment respond well to transmural drainage alone without endoscopic necrosectomy. Protocolized and delayed necrosectomy has been described by several authors as effective and safe, while being associated with a lower number of necrosectomy sessions [49,50]. This approach has been adopted in all RCTs on WON management and is currently recommended by guidelines [4,24,25]. In contrast, a large retrospective study including 271 patients compared immediate (*n* = 69) and delayed (*n* = 202) necrosectomy strategy after LAMS drainage and reported that the immediate strategy group required fewer necrosectomy sessions (3.1 vs. 3.9, *p* < 0.001), with comparable clinical success rate and AEs [51]. Recently, Bang and colleagues conducted a RCT which will probably be of great impact on necrosectomy strategy. The authors randomized 70 patients with infected WON with at least 33% necrotic content to upfront necrosectomy during the index drainage procedure (*n* = 37) or endoscopic step-up treatment (*n* = 33) [52]. The study showed that upfront endoscopic necrosectomy significantly reduced the number of reinterventions to achieve treatment success, significantly increased the proportion of patients with clinical improvement at 72 h and significantly shortened the length of hospitalization as compared with a step-up approach, without increasing adverse events. This study provided high-quality evidence on the advantages of immediate necrosectomy in a subgroup of WON patients (i.e., infected necrosis and high amount of solid material within the collection) and is likely to change the clinical practice. The subsequent question is can we predict which patients will not respond properly to drainage alone and will need an additional necrosectomy? Several studies have tried to address this question, identifying WON size, percentage and extent of the necrosis, persistent organ failure and multiple-organ failure as factors associated with worse outcomes and the need for more aggressive interventions [53,54,55,56]. Recently, Baroud and colleagues tried to standardize WON classification to stratify the disease course according to the complexity of the collection and the need for additional interventions [57]. Seventy-one patients with symptomatic WON were stratified according to the proposed classification, which included the abdominal quadrant distribution (“Q”), the percentage of necrosis (“N”) and the presence of infection (“I”). As expected, a higher QNI score was associated with a higher number of necrosectomies, longer hospital stays, longer mean time to resolution and higher mortality. A higher number of necrosectomies (i.e., ≥2 necrosectomies) was also reported in patients with large WON, hemorrhage, disconnected pancreatic duct syndrome and necrosis pattern in a retrospective study including 104 patients [58]. A retrospective analysis of 101 patients who underwent WON drainage with LAMSs investigated the predictors of clinical failure of endoscopic necrosectomy [59]. Logistic multivariable analysis showed that higher Acute Physiology and Chronic Health Evaluation II score, the extent of pancreatic necrosis and paracolic gutter extension were negatively associated with necrosectomy success. These studies underline the concept that patients with WON are a heterogeneous group with different needs and prognoses. Predicting the complexity could help in optimizing the management (e.g., sending a patient to a center equipped with expertise in endoscopic/surgical necrosectomy or interventional radiology) or in prioritizing necrosectomy if the worst outcome with drainage alone is anticipated. Strikingly, poor interobserver agreement among experts was found in estimating the percentage of solid components in WON, indicating that there is still much work to be carried out to standardize the definition of WON features, to provide reliable and reproducible predictors to be used in clinical practice [60]. Various devices are used to debride necrosis, including grasping forceps, polypectomy snares, nets, Dormia baskets and tripod retrieval forceps (Figure 5). Difficult access to the cavity or abundant and adherent necrotic tissue may increase the complexity of the procedure. A critical point is to avoid damage to retroperitoneal vessels and viable tissue, which could lead to severe AEs. Bang et al. proposed a structured approach to endoscopic necrosectomy, which is performed according to the following steps: (1) debridement using various devices (e.g., snares or rat-tooth forceps); (2) extraction of necrotic debris using forceps, snares or retrieval nets; (3) irrigation using normal saline mixed with hydrogen peroxide, which can be continued in the post-procedure period through naso-cystic catheters (Figure 6) [21,38]. Adopting such a structured algorithm resulted in fewer necrosectomy sessions to achieve clinical success, as reported in a prospective cohort which was compared to a historical retrospective cohort [61]. The advantages of using naso-cystic catheters with irrigation between necrosectomy sessions (usually 500–1000 mL of saline) are uncertain. A retrospective study reported a reduced risk of plastic stent occlusion by threefold (12% vs. 33%, *p* = 0.03), but this advantage has not been confirmed when LAMSs are used for WON drainage instead of plastic stents [62,63]. Some authors have suggested the use of hydrogen peroxide during irrigation to sterilize the cavity and to help the dissolution of the necrosis [64,65]. Retrospective studies reported that the use of hydrogen peroxide was associated with higher clinical success rate and earlier resolution of the collection, with good safety. However, the high number of confounding factors that could influence clinical outcomes limit the conclusions of such studies, and the current lack of high-quality prospective studies does not allow us to firmly recommend in favor or against such a strategy. Similarly, a retrospective study suggested a beneficial effect of proton pump inhibitor withdrawal after WON drainage, hypothesizing that lowering the gastric pH could help dissolve and liquefy the necrosis, potentially reducing the need for necrosectomy [66]. To date, solid data about the efficacy of these strategies are lacking. In selected cases with extensive necrosis, endoscopic necrosectomy can be performed also through a previously placed percutaneous access, after the above-cited “dual modality” drainage approach [35]. To this purpose, large SEMSs to maintain percutaneous access and to allow the entrance of a standard endoscope within the collection have also been used [67,68,69,70]. Percutaneous drainage and necrosectomy can be performed through a single or multiple accesses, which allow extensive irrigation and debridement to be performed, also in anatomical regions away from the gastroduodenal tract, such as the paracolic gutters. The percutaneous tract is usually dilated before the introduction of the endoscope. Thus, necrosectomy can be performed with the usual devices, such as Dormia baskets or polypectomy snares. Endoscopic necrosectomy is burdened by a relevant adverse event rate, related both to the invasiveness of the procedure itself and to the critical condition of the patients in which it is performed. A systematic review including 553 patients from retrospective studies and RCTs reported an overall adverse event rate of 36% [71]. The most common was bleeding (18%), followed by pancreatic fistula (5%) and perforation (4%). Endoscopic hemostasis within the necrotic cavity using hemostatic forceps, self-assembling peptide gel, spray coagulation, glue or clipping has been reported in small case series and case reports [72,73,74,75,76,77]. However, severe bleeding may require radiological intervention with embolization.

## 8. New Devices for Endoscopic Necrosectomy

**Figure 7:**
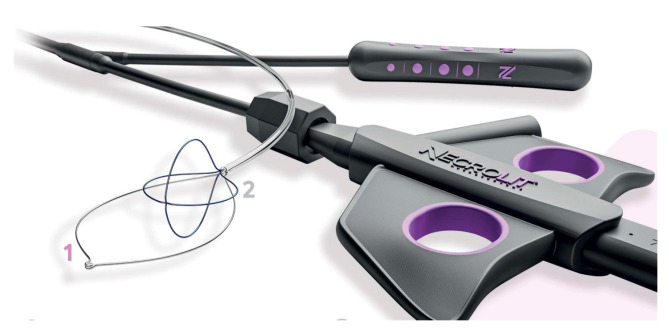
The Necrolit device is composed of a snare (1) and a nitinol basket (2). Image reproduced with authorization of Meditalia s.a.s.

Recently, new devices have been proposed for necrosis debridement, to improve effectiveness, reducing the number of sessions and the timing to WON resolution. The EndoRotor Powered Endoscopic Debridement system (Interscope, Inc., Northbridge, MA, USA) is a dedicated device for resection and removal of the necrotic tissue in WON [78]. The EndoRotor catheter is motorized and provided with a rotating blade, which allows cutting of the tissue and subsequent suctioning with negative pressure. A prospective multicenter study with the 3 mm EndoRotor catheter on 30 symptomatic WON reported an overall successful clearance of 97% (29/30), defined as 70% debris removal [78]. Interestingly, 50% of patients achieved complete debridement in one session, while 73% achieved complete debridement after two sessions (mean number of procedures per patient 1.5). No adverse events related to debridement were reported. More recently, a larger catheter has been proposed, with promising preliminary results. The NecroMax 6.0 (Interscope Inc., Whitinsville, MA, USA) is a 5 mm catheter compatible with 6 mm working channels, or it can be mounted on a standard gastroscope using an EndoRotor catheter guide. A recently published retrospective series reported an overall technical success of 96.7% in 20 patients who underwent 30 necrosectomy sessions [79]. In one case, the procedure could not be performed due to excessive bending of the endoscope, and one patient had perforation, suspected to be device-related. Overall, the study indicated a good feasibility and acceptable safety of the device, even though the data are preliminary. The over-the-scope grasper (Xcavator; Ovesco AG, Tubingen, Germany) is a large transparent plastic cap with distal grasper with a diameter of 14 mm that attaches to the endoscope tip, thus leaving the working channel free to flush and aspirate the debris. The outer caliber allows the passage through a 15 mm LAMS, and the 31 mm opening of the jaws allows the grasping of a large amount of necrosis. A multicenter retrospective study including 37 necrosectomy procedures reported an overall technical success rate of 97%, with a mean of eight pieces (range, 2–25 pieces) of necrosis removed in a mean procedure time of 59 min (range, 15–120 min) [80]. Necrolit (Meditalia s.a.s., Palermo, Italy) is a multiaction device dedicated to endoscopic necrosectomy which allows simultaneous resection and retrieval of the necrotic material. The device is composed of a snare with an ultra-stiff loop to resect the adherent debris and a nitinol basket to retrieve the material (Figure 7). Data about clinical efficacy of this device are awaited.

## 9. Safety of EUS-Guided Drainage

The most frequent AEs related to endoscopic drainage of WON are bleeding, infection, perforation, stent migration/dislodgement and buried stent. Such events are reported in up to 53%, depending on the study design and AE definitions. [38,81,82,83,84,85]. A large retrospective study including 304 patients (151 WON) investigated the adverse events related to PFC drained with LAMSs [86]. The authors reported 79 LAMS-related AEs (overall rate 24.3%), classified as mild in 25.3%, moderate in 68.4% and severe in 6.3% of cases. The AEs were bleeding (22/304; 7.2%), stent migration (20/304; 6.6%), infection (19/304; 6.2%), stent occlusion (14/304; 4.6%), buried stent syndrome (3/304; 0.9%) and occlusion of the pylorus (1/304; 0.3%). In multivariate analysis, WON drainage was a risk factor for AEs compared to pseudocyst drainage (OR, 2.18; 95% CI, 1.09–4.46; *p* = 0.028). Moreover, dilation of the LAMS was protective against AEs (OR, 0.47; 95% CI, 0.22–0.93; *p* = 0.034), probably because dilation protects against the early stent obstruction by the necrotic debris, with subsequent infection [87]. Recently, a nomogram built on a large retrospective series (*n* = 516) of PFC drained with LAMSs identified the injury of the main pancreatic duct, abnormal vessels close to the collection, the use of MTGT and the need for percutaneous drainage as significant predictors of AE occurrence [86]. Bleeding is one of the most feared AEs of WON drainage. It may result from the damage of gastrointestinal wall vessels during drainage or tract dilation, or damage of larger retroperitoneal vessels. Bang and colleagues first suggested that the inner flange of the LAMS could be traumatic against retroperitoneal vessels once the collection collapses, in contrast to the DPSs, which are less traumatic and easily migrate into the gastrointestinal lumen after WON resolution [38,88]. On the other hand, it should be recognized that there is an intrinsic risk of bleeding in WON, which is related to the severity of the inflammatory process [89]. Indeed, a relevant proportion of bleeding can present in the early period after drainage, and thus it is reasonable that multiple factors may play a role in bleeding risk [87]. The placement of DPS coaxial to the LAMS has been proposed as a protective measure against AEs. After fully deploying the LAMS, a DPS is inserted through the LAMS lumen, resulting in the curved ends of the DPS one being in the GI lumen and the other in the collection, with the main axis of the DPS parallel to the axis of the LAMS (Figure 3F). The coaxial DPS could theoretically prevent the traumatic contact of the inner LAMS flange with the retroperitoneal wall after the collapse of the cavity, thus preventing bleeding. Additionally, the DPS could prevent the impaction of both solid debris and food within the LAMS, thus avoiding LAMS occlusion, which is an event that can precipitate cavity infection and sepsis. Retrospective studies have provided conflicting results about the real utility of such a strategy [90,91,92,93]. A recent RCT from Vanek and colleagues reported that DPS groups had a lower global rate of AEs (20.7% vs. 51.5%, *p* = 0.008) and in particular a lower rate of LAMS occlusion (14.7% vs. 36.3%, *p* = 0.042) [94]. However, the primary outcome of the study, which was the need for re-intervention before LAMS removal, was not statistically different among the two groups (29.4% vs. 48.5%, *p* = 0.109), so this study did not provide conclusive evidence on this issue [94,95].

## 10. Conclusions

The impressive technological and cultural advancement of interventional EUS achieved in the last years has placed the endoscopist in a central position in the therapeutic algorithm of WON. A large body of evidence indicates that endoscopic drainage is an effective and safe tool to be used in such patients. Moreover, the availability of Hot LAMS has significantly simplified the drainage technique, thus contributing to the wide spread of the method. Yet, several unresolved issues remain. WON management remains challenging, and a multi-disciplinary approach that involve the endoscopist, interventional radiologist, pancreatic surgeon and specialists in critical-care medicine, infectious disease and nutrition is mandatory to improve the outcome. As previously discussed, efforts must still be made to standardize the definition and classification of WON patients, to tailor the best approach to each case. Individualization of the interventions should lead to the optimal choice of stent to be used for drainage (plastic vs. LAMS), to select the cases where additional drainage routes (i.e., MTGT technique, percutaneous drainage) would be recommended and to define the best timing for endoscopic necrosectomy (index procedure vs. subsequent procedures; scheduled vs. on demand). In this regard, prospective RCTs are currently ongoing [96]. Moreover, endoscopic necrosectomy techniques and devices have evolved much less compared to drainage techniques. Dedicated devices are expected to increase efficacy and safety, while improving the comfort of both patients and endoscopists. Finally, the severity and the relatively high rate of adverse events of WON drainage and necrosectomy highlight once again the need to centralize patients in centers with adequate expertise. The training to conduct such complex procedures is still largely non-standardized, and this represents an issue that will have to be solved in the future to guarantee high performance of the operators and maximize the outcomes.

The way ahead is still long, but the progress achieved so far allows us to look to the future of WON management with great optimism.

## Figures and Tables

**Table 1:** Definition of pancreatic and peripancreatic collections according to the presence of necrosis and timing from the onset of pancreatitis (Atlanta classification).

Pancreatic/Peripancreatic Necrosis	<4 Weeks	>4 Weeks
No	Acute peripancreatic fluid collection	Pancreatic pseudocyst
Yes	Acute necrotic collection	Walled-off necrosis

**Table 2:** Summary of the main prospective studies comparing LAMSs and DPSs for endoscopic WON drainage. LAMS, lumen-apposing metal stent; DPS, double-pigtail stent.

Author, Year (Ref)	Study Design	Population	LAMS	Primary Outcome	Number of Patients	Clinical Success	Number of Proc/DEN Sessions	Length of Hospital Stay (Days)	Adverse Events	Index Procedure Costs	Total Costs
Bang et al., 2019 [38]	Randomized, controlled	Symptomatic and/or infected WON	15 mm	Number of procedures	LAMS = 31 DPS = 29	93.5% (29/31) 96.6% (28/29)	2 ^§^ (2–7) 3 (2–7)	2 (2–7) ^§^ 3 (2–7)	41.9% (13/31) 20.7% (6/29)	USD 12,155 USD 6609 *	USD 53,117 USD 50,132
Boxhoorn et al., 2022 [40]	Comparative, non-randomized	Infected WON	15–20 mm	Need for necrosectomy	LAMS = 53 DPS = 51	NR NR	64% (34/53) 53% (27/51)	43 ^+^ 53	41.5% (22/53) 43% (22/51)	EUR 5056 EUR 2813	EUR 46,860 EUR 53,208
Karstensen et al., 2022 [39]	Randomized, controlled	WON > 15 cm	20 mm	Number of necrosectomies	LAMS = 20 DPS = 22	94.7% (18/20) 95.5% (21/22)	3.1 (3.7) ^+^ 2.2 (3.1)	58 (40–86) 43 (40–67)	5% (1/20) 20% (4/20)	EUR 3839 EUR 2474 *	EUR 39,176 EUR 33,939

WON, walled-off necrosis; LAMS, lumen-apposing metal stent; DPS, double-pigtail stent; DEN, direct endoscopic necrosectomy; ^§^ median (range); ^+^ mean (standard deviation); * *p* < 0.001.

## Data Availability

No new data were created or analyzed in this study. Data sharing is not applicable to this article.
